# Remarks on the Use of Hydrocyanic Acid in Angina Pectoris and Other Diseases of the Heart, and in Bronchitis, Phthisis, and Dyspepsia

**Published:** 1824-05

**Authors:** Michael Ryan

**Affiliations:** London, &c. &c.


ART. III.—*Remarks on the Use of Hydrocyanic Acid in Angina Pectoris and other Diseases of the Heart, and in Bronchitis, Phthisis, and Dyspepsia.* By MICHAEL RYAN, M.D. M.R.C.S. London, &c. &c.

MARY LALOR, ætat. twenty-one, a thin delicate habit, applied to me, July 8th, 1823, in consequence of pain under the left mamma, which, on the slightest exertion, suddenly shoots across to the sternum, inducing a most distressing sense of suffocation, that threatens instant death, and obliges her to stop suddenly. She has dull pain in the left shoulder, extending to the middle of the arm, and sometimes to the fingers of that side, which is always increased during the urgency of that of the chest. Palpitations are troublesome, and are increased with the pain of the sternum by corporeal or mental exertions, and especially by ascending any eminent situation. In ascending the stairs, she is compelled to stop several times, "both to take in breath, and to relieve a great weakness in her knees." These symptoms are of three years' constant duration, are mostly present every day, and for some weeks have been attended with loss of appetite, flatulency, nausea, and other symptoms of dyspepsia. Sleep is disturbed if she lie on the left side, and all her pectoral complaints become aggravated; while she can repose on the right side and back with ease and comfort. Urine is diminished; œdema pedum; bowels confined; catamenia regular; pulse 120, small, quick, but regular; and respiration very laborious during the paroxysm. She thinks she cannot recover, her sensations are so distressing. Leeches and blisters were applied to the left side, and many internal remedies administered, by my late much respected namesake, but with only temporary advantage.

The dyspepsia was relieved by the ordinary remedies on August 9th, but the pain in side and sternum continued unabated.

℞. Tart. Antimonii, drach. duas;

Axungiæ Porcinæ, unc. unam; fiat ung. cujus pauxillo picetur pars lateris affect. M. n. donec erumpant papulæ.

℞. Tinct. Digitalis, Vini Colchici, āā drach. unam;
Acidi Hydrocyanici, guttas viginti. M. sit mistura, cujus sumat. gt. xx. ex aquæ cyatho, bis de die.

September 12th.—Has used the remedies, but did not take the drops so often as directed, as they frequently induced head-ach and nausea. Pain in the chest is removed; can now ascend any elevated situation, without the least inconvenience. She ascribes her cure to the last medicine, which she has repeated: it first removed the palpitations, next the pain in the region of her heart, and ultimately that of the sternum. Her gums are slightly inflamed, and slight salivation is present, which she ascribes to the influence of the medicine: she is otherwise free from complaint.

November 12th.—Pulse 88, full and regular; appetite and general health very good.

January 24th, 1824.—Continued free from complaint since last report, until this day, when, being about a mile distant from her residence, was suddenly sent for; she ran quickly,—the pain in the left side and sternum recurred, and her chest-complaint returned. Pulse 128, very quick, small, but regular. No pain of left arm; but can scarcely move without aggravating all her complaints.—Repetatur mistura Acid. Hydrocyanici, die 9 August præscript.

February 8th.—Is much relieved from the pain; appetite good.—Con. mistura.

12th.—Pain in side and chest relieved, and can now use a considerable exertion without inducing a return of her complaints.

March 1st.—She reports herself free from complaint.

From the symptoms of this patient, I have little hesitation in asserting that the affection of the heart was idiopathic, and what is called angina pectoris; while that of the stomach was only symptomatic of the former. The digitalis was exhibited to regulate the circulation; the colchicum, on account of the symptoms of incipient hydrothorax; and the hydrocyanic acid, to allay the irritation of the organs of respiration and circulation. This acid is said to destroy life by extinguishing nervous energy, from the fact that animals submitted to its influence breathe freely for hours, the circulation of the blood also remaining natural, though no trace of sensibility can be found. Hence, from analogy, the belief arose that it would be useful in allaying excessive sensibility and irritation of the organs of respiration and circulation. From such reasoning Professor Brera, in Italy, was induced to exhibit it in inflammation of the lungs so early as 1813, and he observed that the violence of the disease quickly abated. Dr. Granville, of London, was the first who introduced this valuable remedy into British practice in the treatment of "pulmonary consumption, and in other diseases of the chest, as well as in several complaints attended with great irritation and acute pain." This judicious physician remarks, that "the acid, when administered in cases of vascular or other excitement, has an immediate influence over the nervous system; it gradually diminishes irritability, checks a too-rapid circulation, and calms many of the symptoms of fever. If dry cough be present, it promotes expectoration, and often stops the cough itself.*" It is said to relieve cough from various causes, hectic fever, asthma, chronic inflammation in various organs, pneumonia, pleurisy, catarrh, pertussis, bronchitis, menorrhagia and dysmenorrhœa, hemoptysis, and some nervous diseases, as those of the stomach, and pulmonary consumption. Magendie in France, Brera in Italy, Behr and Helmekin in Germany, Dr. Granville in England, and Dr. Nancrede in America, strongly recommend hydrocyanic in pulmonary consumption; whilst Drs. Elliotson and Macleod are against it.† Dr. Macleod was one of the first who called in question the efficacy of this remedy in phthisis and chronic bronchitis of elderly persons, "because the relief it afforded in such cases was inferior to what was produced by more common remedies."‡ He was the first who observed it produced salivation and soreness of the gums; neither did he find it useful in dyspepsia.§

Dr. Granville recommends the use of this acid in angina pectoris,‖ from which suggestion I was induced to try it in the above case; although I am aware that Dr. Darwall, of Birmingham, found it useful in a disease of the heart, accompanied with pain, palpitation, and expectoration;¶ but certainly I am unacquainted with the publication of any case so strongly marked as that of my patient, in which the acid had been used, if I except the recommendation of Dr. Granville. I first administered it in August last, it was effectual in September; while Dr. Macleod had tried it "in a case with well-marked symptoms of enlargement of the heart, attended with pain, palpitation, and dyspepsia," in November, and published his observations on it in December last; of which I could have no previous intimation in the preceding August: neither were the cases nearly analogous, though the ideas of both physicians seem to be ori-

* Dr. Granville *on Hydrocyanic Acid*, London 1820; also *London Med. and Phys. Journal*, 1821, vol. xlvi. p. 45.
† *Op. cit.* January 1822, vol. xlvii. p. 31.
‡ *Id.* vol. xlvi. p. 359.
§ It will be seen, by Dr. Macleod's paper in this Journal (December 1823,) that he regards the prussic acid as useful in *particular forms* of dyspepsia.—Edit.
‖ *Id.* December 1821, p. 359.
¶ *Id.* November 1822, vol. xlviii. p. 447.

ginal. Again, Dr. Heller had used the acid in phthisis without success, and in six cases of disease of the heart, with palpitation, in August 1823;* but the first account I have seen of these cases was in January 1824.† The writers, then, against the efficacy of the acid in phthisis, since 1821, are Dr. Darwall, Dr. Paris,‡ and Dr. Macleod.§

In the best marked case of hereditary phthisis, in a subject born in a tropical climate and residing in this, which I have ever seen or read of, the cough and hectic symptoms were removed, the expectoration gradually diminished, and the appetite improved, by the use of the acid; yet this young lady gradually continued to waste: there was no discharge whatever to account for such emaciation; her respiration was free and easy until she expired. Another sister and the father, while dying of this dreadful disorder, had cough so urgent, the respiration so laborious, in the last stages of the disease, that the very approach of the attendants towards the bed caused almost a sense of suffocation; the hectic symptoms were most distressing; but the acid was not in use at the time. I have tried it in coughs, bronchitis in old persons, in asthma, and diseases of the heart; and in all I deem it a most valuable remedy. I have given it also in a few cases of dyspepsia, but certainly with little advantage.

For further remarks on this acid, see the following essays:—Murray on Hydrocyanic Acid, *London Medical and Physical Journal*, January 1823; Review of Dr. Paris's Pharmacologia, *id.*; Experiments instituted on it at Florence, *id.*; Dr. Macleod on its Use in Diseases of the Heart and Stomach, *id.* December; M. Pessina on making Prussic Acid, *op. cit.*

Hydrocyanic acid readily undergoes decomposition, even if it touch the cork of a phial in which it is enclosed; it falls to the bottom of the vessel, if mixed with water, and should be always shaken previous to use under such circumstances. It should be combined with mucilage and syrup in the most common form, and the addition of digitalis will be found very beneficial in diseases of the heart. On the whole, I agree with those who think this acid a valuable and excellent remedy in the cure of the above-cited diseases; and I think the profession greatly indebted to Dr. Granville for its introduction into the United Kingdom as a medicine, as well as for the many other valuable suggestions and improvements which he so largely contributed to medical science, through the medium of this excellent Journal.

Angina pectoris is an organic disease of the coronary arteries of the heart, inducing a deficiency of nutriment in its parietes;

* *Revue Medicale.*
† *London Med. and Phys. Journal,* January 1824.
‡ *Pharmacologia,* and *London Med. and Phys. Journal,* February 1823.
§ *Op. cit.* December 1823.

and hence it is astonishing that such disorganisation could admit even of palliation: yet alleviation has often taken place under such circumstances, and even where great disorganisation had been found on dissection after death. Though this affection must have existed in all ages, the ancient Greek and Latin physicians are silent on the subject. Erasistratus certainly describes symptoms scarcely to be applied to any other disease;* and Morgagni described enlargement of the heart and its large vessels, even ossification of the coronary arteries.†

The first account of angina pectoris was communicated by the late Dr. Smyth, of Dublin, to Dr. M'Bride, the sufferer from which applied to the former in 1760, and laboured under exquisite constrictory pain of the sternum, extending to each of his arms, as far as the insertion of the deltoid muscle; extreme anxiety; laborious breathing, strangling, and violent palpitation of the heart, with a most irregular pulse. The paroxysms were frequent, and he scarcely escaped a day, for six or seven years, without one. They were excited by exertions of the mind or body; he could not go up stairs; and his disease commenced at seventeen.‡ We have the testimony of Mr. Burns, who states this disease may occur in early life.§ I should observe that Dr. Smyth had seen eight other cases, in two of which the patients dropped dead suddenly.

An essay was read by Dr. Heberden, before the Royal College of Physicians, London, in July 1768, who stated that the sense of strangling, the seat of it, and the anxiety with which it was attended, may make it not be improperly called angina pectoris.‖ He published two other cases in the Medical Transactions, vol. iii. 1772; one of which Mr. Hunter dissected, and in which Dr. Jenner assures us that the state of the coronary arteries was not attended to.¶ The second case was that related by Dr. Wall, of Worcester, and there was no attention paid to the coronary arteries. Indeed, Dr. Heberden was of opinion, in 1774, "that malformation of the parts was not the cause of this disease."** Dr. Fothergill published two cases, (Med. Obs. and Enq. vol. v. 1776,) in the first of which there was no allusion to the coronary arteries; but in the second (the case of H. R. esq.) Mr. Hunter found these arteries "one piece of bone;" although he did not deduce it as the cause of

---

* Cœlius Aurelianus *de Morb. Diuturnis*, lib. ii. cap. l. p. 317.

† Morgagni *de Sedibus et Causis Morb.* lib. ii. Epist. 26, art. 31; *Id.* lib. ii. Epist. 24, art. 17. Also Haller, *Elements of Physiology*; Bellini *de Urinis et Pulsibus*, 4to. 1698, p. 616; Crellius *de Arteria Coronaria instarossis in duzata*, 1740.

‡ *Med. Commentaries*, 1777, vol. v. p. 97; also M'Bride's *Pract. Physic*, 1777.

§ Burns *on Diseases of the Heart*, p. 147.

‖ *Med. Trans. Coll. Phys.* vol. ii. p. 59.

¶ See Parry *on Syncope Anginosa.*

** *Med. Commen.* vol. iii. 1775, p. 181.

the disease for some time afterwards.* It is a curious circumstance how that great man discovered the cause of a disease, to which it was his fate to fall a victim seventeen years afterwards.

Dr. Black, of Newry, was consulted about a case of this disease in March 1792, and sent an essay on it, and an account of the morbid dissection, to the London Medical Society, which was read March 1794; and this dissection was considered, by the late Dr. Percival, of Manchester, "the most satisfactory and important of which he had any knowledge;" and the Society voted Dr. B. a silver medal, as a mark of their approbation.† Thus it appears that Mr. Hunter was the first, and Dr. Black the next, who attended to the state of the coronary arteries on dissection.

Dr. Parry, of Bath, published an erudite essay on this disease in 1799, and states that he read an essay on angina pectoris, in 1788, before a Medical Society at Rodborough, of which the late ingenious Dr. Jenner was a member; and that Dr. J. suggested that the disease probably arose from some morbid change in the structure of the heart; and hence Dr. Parry wished to prove, from the date of Dr. Black's essay in 1792, "that it had no share in producing the conclusion of Dr. Jenner or himself, as to the cause of angina pectoris, in 1788." It is quite as evident that Dr. Black derived no suggestion as to the cause of the disease from the opinions of Drs. J. and P., which were first published in 1799, seven years after the former had published his opinions on the subject. Dr. Parry also asserts, that the cases of angina pectoris, noticed by Drs. Smyth and M'Bride, were only instances of severe palpitation, which must be frequently observed by every extensive practitioner. Now, both these gentlemen were deservedly at the head of extensive practice, in the second city of Great Britain, for many years, and were, perhaps, as little liable to mistake as any physician who succeeded them; and we should remember that the symptoms of the affection under consideration, as laid down by them, have been always received as authentic by every writer since their time, even to the present day. I do think, therefore, with great respect for the opinions of Dr. Parry, that such proofs are insuperable, and perfectly conclusive.

Dr. Black published a second case of the disease, in 1796, in the Memoirs of the Medical Society of London; and the late Dr. Curry published two additional cases, in the Medico-Chirurgical Transactions, vol. vii. Dr. Blackall added an Appendix to his work on Dropsies, on angina pectoris. For further information, see the following essays:—Hill on Angina Pectoris, *London Medical and Physical Journal*, vol. iv. 1800, page

* Reeder *on Diseases of the Heart*, p. 54.

† *Memoirs of the London Medical Society*, vol. iv.

30; CAPPER on ditto, *op. cit.* p. 222; GOODWIN, *id.* vol. vi. 1801, p. 320; PATTERSON, *id.* vol. x. 1803, p. 63; WELDON, *id.* vol. xvi. 1806, p. 487; RING, *id.* vol. xvii. 1807, p. 9; REID, *Transactions of the College of Physicians, Dublin*, 1818, vol. i. p. 295.

*Kilkenny, Patrick-street; March* 8*th*, 1824.

